# Experiments Relative to the Analysis of Opium

**Published:** 1803-07-01

**Authors:** 

**Affiliations:** Apothecary at Erfurt


### EXPERIMENTS *relative to the* ANALYSIS *of* OPIUM; *by* Mr. BUCHHOLZ, *Apothecary at Erfurt.*

[ Continued from Vol. ix. p. 403. ]

EXP. XXIV. ON mixing this spirituous solution with an equal portion of water, a black matter was immediately precipitated, and the solution remained clear; but on adding another part of water, it became turbid; and when the proportion of the water to the spirit of wine was as 8 : 1, a limy and pulverulent matter was separated, while the mixture lost its colour almost entirely.

EXP. XXV. Ninety grains of the residuum of opium, from which all soluble matter had been extracted by water and spirit of wine, were infused twice successively with two ounces of vitriolic ether. The residuum being carefully dried, weighed 67 grains.

EXP. XXVI. Forty-six grains of resinous particles, obtained from a portion of opium, which I had some time before analysed, were infused with two ounces of alcohol, and boiled for a quarter of an hour, by which means the alcohol became strongly tinged. Having repeated the experiment, I separated 5 grains of a light brown pulverulent matter, besides which the solution of the resin yielded one grain more of the same substance, which treated together with 30 parts of vitriolic ether, proved to be insoluble, and likewise when boiled with 50 parts of distilled water, 100 parts of spirit of wine dissolved not quite two parts of this matter, the residuum receiving a lighter colour, and being soluble with still greater difficulty.

EXP. XXVII. This matter was easily soluble in concentrated nitric acid when heated, whereby a similar smell was disengaged, as when animal parts are treated with this acid. Water, however, again separated it. It was likewise easily dissolved in caustic lye; the solution had a dark brown colour, and emitted the smell of animal gluten: it separated itself by the addition of some nitric acid.

EXP. XXVIII. A solution of iron rendered the solution of this matter in alcohol somewhat darker, but did not blacken it. From these experiments it seems to result, that this particular matter consisted of a combination of gluten with resina.

EXP.

Exp. XXIX. The above-mentioned 67 grains (Exp. 25) were treated with 160 gr. of caustic kali, and 4 ounces of distilled water in a boiling heat, during which operation a matter was disengaged, smelling like an animal substance when ready to putrify. The whole, except 10 grains of a lamellous, or fibrous matter, was dissolved. The solution being filtrated, was of a brown colour.

Exp. XXX. Having sufficiently edulcorated the matter which remained on the filtrum, I gradually saturated the kali with muriatic acid, by which means I obtained a brown viscous precipitation, weighing 5 grains when dry. Thrown on living coals, it emitted the smell of horn; and burnt in a crucible, it left a glossy coal, which could be with difficulty calcined, and after which some calcareous earth remained.

Exp. XXXI. I evaporated the solution in which the remaining 52 grains were contained, to dryness, by which means I obtained muriat of kali, and the blackish viscous matter, smelling like burnt animal gluten, and being soluble both in water and spirit of wine; whence it appears that the greatest part consisted of animal vegetable matter or gluten.

Exp. XXXII. One hundred grains of the residuum of the watery extract, (Exp. 21) were incinerated in a clean crucible, from which a reddish mass remained, weighing about 0,035, which boiled in two ounces of water for a quarter of an hour dissolved, 0,01 excepted. I boiled this grain with 3 drops of concentrated nitric acid, and half a drachm of distilled water, by which it was dissolved. On adding to it a portion of a solution of muriat of barytes, it was troubled, and also by the addition of oxalat of kali, &c. from which it appeared to contain gypsum, aluminous earth, and iron.

Exp. XXXIII. The watery solution, containing the 0,025 saline parts was evaporated by a gentle fire, by which 2 grains or 6,02 regular crystals of sulphat of kali were obtained, the rest consisted of sulphat of lime. No free kali could be traced.

Exp. XXXIV. Sixty grains of the residuum from the tinctura thebaica were boiled with 16 ounces of very pure vinegar to 8 ounces, by which 10 grains were dissolved. The residuum of 50 grains was again boiled with 20 ounces of vinegar; but though this was continued for above an hour, nothing had been dissolved. It contained the gluten and the Koutshoock matter. I had undertaken this experiment ment for the purpose of ascertaining the opinion of some chemists, that the residuum remaining after the spirituous and watery extraction, is soluble in vinegar, which however seems not to coincide with these experiments. In order to examine which menstruum is the most proper for the solution of opium, according to the knowledge of this substance acquired by the foregoing experiments, I made the following trials.

Exp. XXXV. Two hundred and fifty grains of opium, cut into small pieces, were digested with 4 ounces of alcohol, and the same quantity of distilled water, for 24 hours, and at last boiled for two hours, whereby the liquor was deeply tinged. I poured the mixture on a filtrum, adding to it a similar mixture of alcohol and water, as long as it run through coloured. I mixed the residuum with one ounce of alcohol and the same quantity of water, and boiled it together for half an hour; the residuum weighed 45 grains, after being edulcorated and dried; whence it appears that all resinous particles are contained in this solution, otherwise the residuum would have amounted to 68 or 69 grains, which was its quantity when I had extracted the opium by water only. It is probable, that the saponaceous and gummous particles contained in the spirituous solution promote the solution of the resin in weak spirits of wine, as it appears from the above described experiments, how difficult it was to extract the resinous particles entirely by alcohol.

Exp. XXXVI. Two hundred and fifty grains of the best opium being pulverised, were digested for 12 hours with 8 ounces of Malaga wine, and afterwards gently seethed for two hours, filtrated, and the residuum edulcorated with the same wine and some water. The experiment was repeated with the residuum and three ounces of Malaga, but the liquor seemed not much to have been impregnated with opium. The rest, which was not soluble, amounted to 54 grains. Both the spirituous as well as the vinous solution had, after some days, deposited two grains of gluten.

### *Results of this Analysis of Opium.*

1. Opium contains the following constituents, and in the subjoined proportion.

*a* According to the experiments 1, 5, 6, 16, &c. in 500 grains, 178 gr. of extract, both soluble in alcohol and water, or saponaceous extract.

*b*. According to the experiments 1, 5, 6, 16, &c. 152 grains of gum, only soluble in water.

*c*. Ac-

*c.* According to the experiments 8, 9, 10, &c. in 500 parts, 45 parts of which are only soluble in alcohol.

*d.* The eighth part of these 45, seems to be resin, intimately mixed with gluten, according to the experiments 22, 23, 24, 26, &c.

*e.* Opium contains, according to the experiments 12, 13, 15, 25, in 500 parts, 24 parts of Koutshoock matter.

*f.* According to the experiments 29, 30, &c. from 50 to 60 grains of vegeto-animal matter, or gluten, in 500 grains, and some ligneous matter.

*g.* According to the experiments 20, &c. 30, &c. about 2 or 3 per ct. of sulphat and muriat of kali, sulphat of lime, some aluminous earth.

*h.* According to the experiments 1 and 2, volatile narcotic matter = g.

These constituents, calculated according to 100 parts, will make,

| | | |
|---|---|---|
| 1 | Saponaceous matter | 35,60 |
| 2 | Gum — — | 30,40 |
| 3 | Resinous particles | 9,— |
| 4 | Koutshoock matter | 4,80 |
| 5 | Glutinous matter | 11,40 |
| 6 | Ligneous substance | 2,— |
| 7 | Loss — — | 6,80 |
| | | 100 |

2. The saponaceous matter seems to render the solution of the resinous particles of opium much easier even in weak spirit of wine.

3. The best menstruum for opium is a mixture of equal parts of alcohol and water, by which all the soluble particles of opium are extracted and united. Hence it appears, that the prescription of the Edinburgh dispensary for preparing tinctura thebaica is the most proper, and the best calculated for extracting all the active parts of opium.

4. According to experiment 3, it seems probable that the volatile particles which affect the organs of smell, do not contain the principal virtue of opium; but it seems rather to reside in the other, particularly in the resinous particles, the taste of which is very acrid and penetrating.

5. The fear of impairing the virtue of opium by boiling it with different menstrua, is for the most part exaggerated and groundless.

6. Opium contains no essential oil.

7. Astringent

7. Astringent matter or Gallic acid, does not belong to the constituents of opium.

The results of this examination of opium, although they may seem not quite satisfactory, are certainly entitled to the attention of every chemist and physician.

